# Retention of Bioflx, Zirconia, and Stainless Steel crowns using two different luting cements in primary molars: an in vitro study

**DOI:** 10.1186/s12903-025-06671-2

**Published:** 2025-08-15

**Authors:** Nour Gamal Morsy, Karin ML Dowidar, Mona Mohy El Din, Salma Abolgheit, Dina Aly Sharaf

**Affiliations:** 1 Paediatric Dentistry and Dental Public Health Department, Faculty of Dentistry, Alexandria University, Alexandria, Egypt; 2 Dental Biomaterial Department, Faculty of Dentistry, Alexandria University, Alexandria, Egypt

**Keywords:** Full coverage crowns, Luting cements, Crown retention, Retention force, Stainless steel crowns, Bioflx crowns, Zirconia crowns

## Abstract

**Background:**

Different crown types are used for the full-coverage restoration of primary teeth with extensive caries. Bioflx crowns are hybrid polymer-resin crowns designed to combine the benefits of Stainless Steel and Zirconia. The longevity and clinical efficacy of dental restorations are significantly affected by crown retention. We aim to assess the retention of Bioflx, Zirconia, and Stainless Steel crowns using two different luting materials.

**Methods:**

Fifty-four (*n* = 54) freshly extracted mandibular second primary molars were divided into three main groups (*n* = 18): Bioflx crowns, Zirconia crowns, and Stainless Steel crowns (SSCs). Each group was further subdivided into two subgroups (*n* = 9), using either Conventional Glass Ionomer luting cement (GIC) or Resin Modified Glass Ionomer luting cement (RMGIC). A retention test was performed to determine the retentive force required for crown removal after the samples underwent 2,000 cycles of thermocycling. A stereo light microscope was used to examine the debonding failure. Data were analysed using two-way analysis of variance (ANOVA) and Pearson’s chi-square test.

**Results:**

Stainless Steel crowns exhibited the highest retention in both subgroups (GIC = 307.44 ± 53.58 N, RMGIC = 324.11 ± 52.04 N). Bioflx crowns outperformed Zirconia crowns (Bioflx: GIC = 138.11 ± 30.87 N, RMGIC = 218.11 ± 34.61 N; Zirconia: GIC = 35.50 ± 5.14 N, RMGIC = 131.78 ± 11.91 N). All differences between the GIC and RMGIC subgroups were significant (*p* < 0.001), except for Stainless Steel crowns (*p* = 0.5). RMGIC had greater retention values than GIC in all groups, and the difference was significant (*p* < 0.001), except for the SCCs group (*p* = 0.5).

**Conclusions:**

Stainless Steel crowns showed the highest retention across all tested luting cements, followed by Bioflx crowns, which exhibited superior retention compared with Zirconia crowns. RMGIC showed superior retention compared with GIC.

**Supplementary Information:**

The online version contains supplementary material available at 10.1186/s12903-025-06671-2.

## Background

Selection of an appropriate restorative technique is crucial for achieving optimal outcomes in paediatric dentistry [1]. The American Academy of Paediatric Dentistry recommends the use of prefabricated full-coverage restorations for primary teeth with large or multi-surface carious lesions, emphasising their effectiveness in ensuring long-term treatment success [2]. This treatment option offers strong, enduring protection and helps prevent caries from recurring [3, 4].

Stainless Steel crowns (SSCs) remain the gold standard in paediatric dentistry for full coronal coverage, offering significant benefits such as reliability, durability, minimal tooth preparation, improved retention, and low cost [5]. However, Stainless Steel crowns have several notable drawbacks. The metallic appearance may negatively impact a child’s self-image and parental approval, making aesthetics a critical factor in treatment planning [6, 7]. In addition, surface roughness can promote bacterial adhesion and biofilm accumulation, thereby increasing the risk of periodontal disease. Moreover, metal ions, such as nickel, when released under certain conditions, may cause hypersensitivity in some patients [8, 9].

In the past few years, more aesthetic full-coverage restorative options have been introduced in paediatric dentistry. Prefabricated Zirconia crowns have grown in popularity because of their high clinical acceptance and aesthetic satisfaction among parents and children [10]. Zirconia crowns offer several advantages, including tooth-like colour, translucency, and high polishing [4]. They are also biocompatible and cause minimal gingival irritation, which are desirable properties in aesthetic dental materials [11, 12]. However, Zirconia crowns require more aggressive tooth preparation than Stainless Steel crowns [4, 13]. As their margins cannot be adjusted, Zirconia crowns rely entirely on the retention provided by the luting agent [14, 15]. Moreover, Zirconia crowns can cause enamel wear of the opposing tooth [16]. The limitations associated with Zirconia and SSCs have prompted the exploration of alternative aesthetic restorations. More recently, preformed Bioflx crowns have represented the latest advancements in the category of aesthetic crown options.

Bioflx crowns are designed to combine the flexibility, durability, and self-adaptability of SSCs with the aesthetic appeal of Zirconia crowns [17]. Bioflx crowns consist of high-impact hybrid radiopaque polymer resin [18]. They are monochromatic tooth-coloured crowns free of metals or bisphenol A-glycidyl methacrylate. Bioflx crowns offer notable clinical advantages over both Zirconia and Stainless Steel crowns. They cause less wear on opposing natural dentition than Zirconia crowns [19, 20]and demonstrate greater wear resistance than Stainless Steel crowns [21]. Their ability to self-adapt by forming occlusal dimples in high-contact areas enhances functional efficiency [21].

The retention of crowns is essential for preserving the functionality and structural integrity of primary molars, maintaining proper space for the eruption of successors, speech development, and supporting mastication [2, 22]. Successful retention requires both mechanical and chemical methods. Mechanical retention is achieved through proper crown sizing, contouring, and crimping, whereas luting cements that adhere the crown to the tooth achieve chemical retention, ensuring stability until natural exfoliation [22–25].

Cement failure is the primary cause of crown dislodgement, making the selection of an appropriate luting agent critical for long-term clinical success [26]. An ideal luting cement must fulfil several key requirements, such as effective adhesion to both the crown material and the tooth, high tensile and compressive strengths to withstand occlusal forces, and biocompatibility to support periodontal health and maintain a secure marginal seal. Additional desirable characteristics include low solubility in oral fluids, low viscosity for easy handling, minimal film thickness for precise crown adaptation, and a balance between sufficient working time and rapid setting [27, 28].

It is recommended to use RMGIC and GICs for the cementation of paediatric crowns, especially Bioflx crowns [29]. Numerous studies have tested the retention of SSC, Zirconia, and Bioflx crowns using different luting cements [26, 30–35]. These studies have primarily used acrylic and epoxy dies. The current literature offers limited evidence comparing the retention of Bioflx, Zirconia, and Stainless Steel crowns on natural teeth. Therefore, this study aimed to examine the retention of Bioflx, Zirconia, and Stainless Steel crowns using two different luting agents: GIC and RMGIC.

The null hypothesis was that there would be no statistically significant difference in retention among the various crown types and that the type of cement used would not affect retention within each crown group.

## Methods

The modified CONSORT checklist was used as a guideline in the design of this study [36]. This study was carried out at the Department of Dental Biomaterials and Department of Paediatric Dentistry and Dental Public Health, Faculty of Dentistry, Alexandria University, Egypt, with the approval from the Ethics Committee of the Faculty of Dentistry, Alexandria University, Egypt (0843-01/2024) (IRB No: 00010556-IORG:0008839).

A sample size of 54 crowns was determined based on a 5% alpha error and 80% study power. The mean ± SD retention values were 337 ± 137.1 N for Stainless Steel Crowns and 106.5 ± 77.7 N for Zirconia crowns [33]. Assuming that Bioflx crowns have the same retention values as Zirconia crowns, the sample size was calculated for independent means using the highest SD = 137.1 to ensure power. A sample of seven crowns was required, which was increased to nine crowns to compensate for laboratory processing errors.

### Grouping

Two hundred freshly extracted mandibular second primary molars were collected after normal exfoliation or extraction for orthodontic reasons. Inclusion criteria were intact mandibular second primary molars free from caries, absence of cracks as detected under a magnifying lens, and mesiodistal (MD) widths ranging from 10.0 to 10.5 mm, corresponding to the dimensions suitable for the size 4 crowns used in this study. The exclusion criteria included primary molars with restorations, caries, developmental defects, or cracks. One hundred forty-six teeth were excluded: 116 exhibited MD widths outside the required range of 10.0–10.5 mm, 16 had occlusal caries, 11 had proximal caries, and 3 presented with cracks detected under magnification. A final study sample of 54 mandibular second primary molars was included (*N* = 54).

**Fig. 1:**
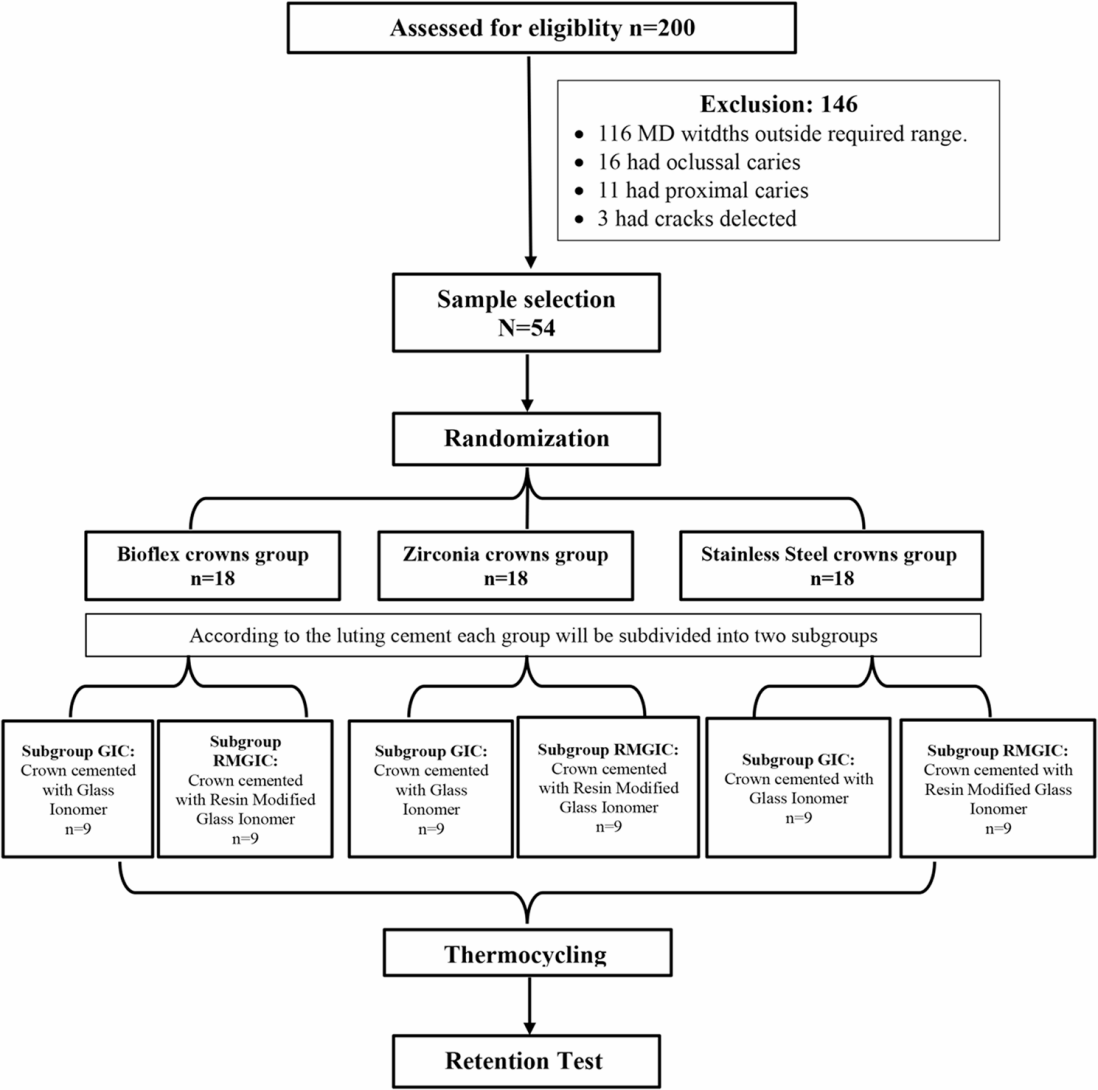
Study Flow Chart

Each tooth was first assigned a number and labelled accordingly on acrylic resin blocks. An independent researcher used these identification numbers to perform random allocation into three main groups using a computer-generated randomisation list: Bioflx crowns (*n* = 18) (NuSmile, Houston, US), Zirconia crowns (*n* = 18) (NuSmile, Houston, US), and SSCs (*n* = 18) (3 M ESPE, St. Paul, Minn, USA), which represented the control group. Each group was further subdivided into two subgroups (*n* = 9) using either Conventional Glass Ionomer luting cement (GIC) (Fuji I, GC Corporation, Tokyo, Japan) or Resin-Modified Glass Ionomer luting cement (RMGIC) (FujiCem 2, GC Corporation, Tokyo, Japan). (Fig. 1).

### Specimen preparation

Initially, a hand scaler was used to clean the teeth, followed by rinsing with water to eliminate any tissue residue. The teeth were kept at room temperature for 1 month in sterile 0.1% thymol solution [27]. They were then encased in cylindrical acrylic resin blocks with a radius of 2 cm and placed 1 mm beneath the cemento-enamel junction.

### Tooth preparation

Before tooth preparation, a Boley gauge was used to measure the MD width of each mandibular second primary molar (mm). Only teeth with MD widths ranging from 10.0 to 10.5 mm were included in this study. Two researchers independently verified the selection process. All crown preparations in all the groups were carried out following the manufacturer’s guidelines [37]. To ensure procedural consistency, tooth preparation was performed by a single trained operator.

The occlusal surfaces of the teeth that received Bioflx crowns were reduced by 1.5–2 mm using a 69 L flame-shaped diamond bur and maintaining the cuspal inclines. Slicing of the proximal surfaces was performed using a tapered fissure bur converging towards the occlusal surface. All sharp lines and point angles were smoothed using a tapered fissure bur. Lingual convexities and buccal bulges were reduced while maintaining a snug fit [37].

The occlusal surfaces of the teeth prepared for Zirconia crowns were reduced by 1.5–2 mm, following the natural contours, using a flame-shaped diamond bur, following the natural contours. In addition, the tooth structure was circumferentially reduced by 1–1.5 mm.

Teeth prepared for Stainless Steel crowns had the occlusal surface reduced by 1–1.5 mm, following the normal contour of the tooth. Using a tapered fissure bur, the proximal surfaces were sliced, converging in the direction of the occlusal surface. All sharp lines and point angles were removed using a high-speed No. 69 L bur.

A digital Boley gauge was used to measure the occlusocervical and MD dimensions of each tooth before and after preparation to determine the amount of tooth structure removed and standardise the tooth preparation across each group.

### Crown cementation

Before cementation, all specimens were washed and dried, but were not allowed to desiccate. In the GIC subgroup, crowns were luted using Fuji I (GC Corporation, Tokyo, Japan), following the manufacturer’s recommendations. The capsule was shaken, activated, and placed in an amalgamator for 10 s. The luting agent was dispensed onto the specimen using an Applier [38]. In the RMGIC subgroup, the specimens were luted with FujiCem 2 (GC Corporation, Tokyo, Japan) in accordance to the manufacturer’s recommendations. The cartridge was checked for proper flow, and a minimal amount of paste was dispensed onto a paper pad after attaching the mixing tip. Subsequently, a mixing tip was used to apply the paste directly to the crowns [39].

After mixing the luting cement, the crowns were positioned correctly on the prepared teeth using finger pressure and supported for 10 min by a 5 kg static force with a special loading apparatus [40]. After excess cement removal, all specimens were immersed in artificial saliva with the pH adjusted to 7.0–7.4 using a pH meter. The artificial saliva was prepared by dissolving 0.220 g of calcium chloride, 1.68 g of sodium bicarbonate, 1.07 g of sodium phosphate, and 2 g of sodium azide in 1 liter of distilled water. The samples were stored in the prepared solution and incubated at 37 °C for 24 hours [41].

### Aging by thermocycling

Thermal aging was conducted using a custom-made thermocycling machine (El-Abbasy F.A; specially designed in the Biomaterials Department, Faculty of Dentistry, Alexandria University). The crowns were immersed in a water bath chamber and subjected to 2,000 cycles ranging from 5 °C to 55 °C, with a dwell time of 30 s and a transfer time of 10 s, mimicking the oral cavity environment. This thermocycling process simulates approximately 2 years of intraoral exposure, with each cycle corresponding to the duration of one meal [42].

### Retention test

**Fig. 2:**
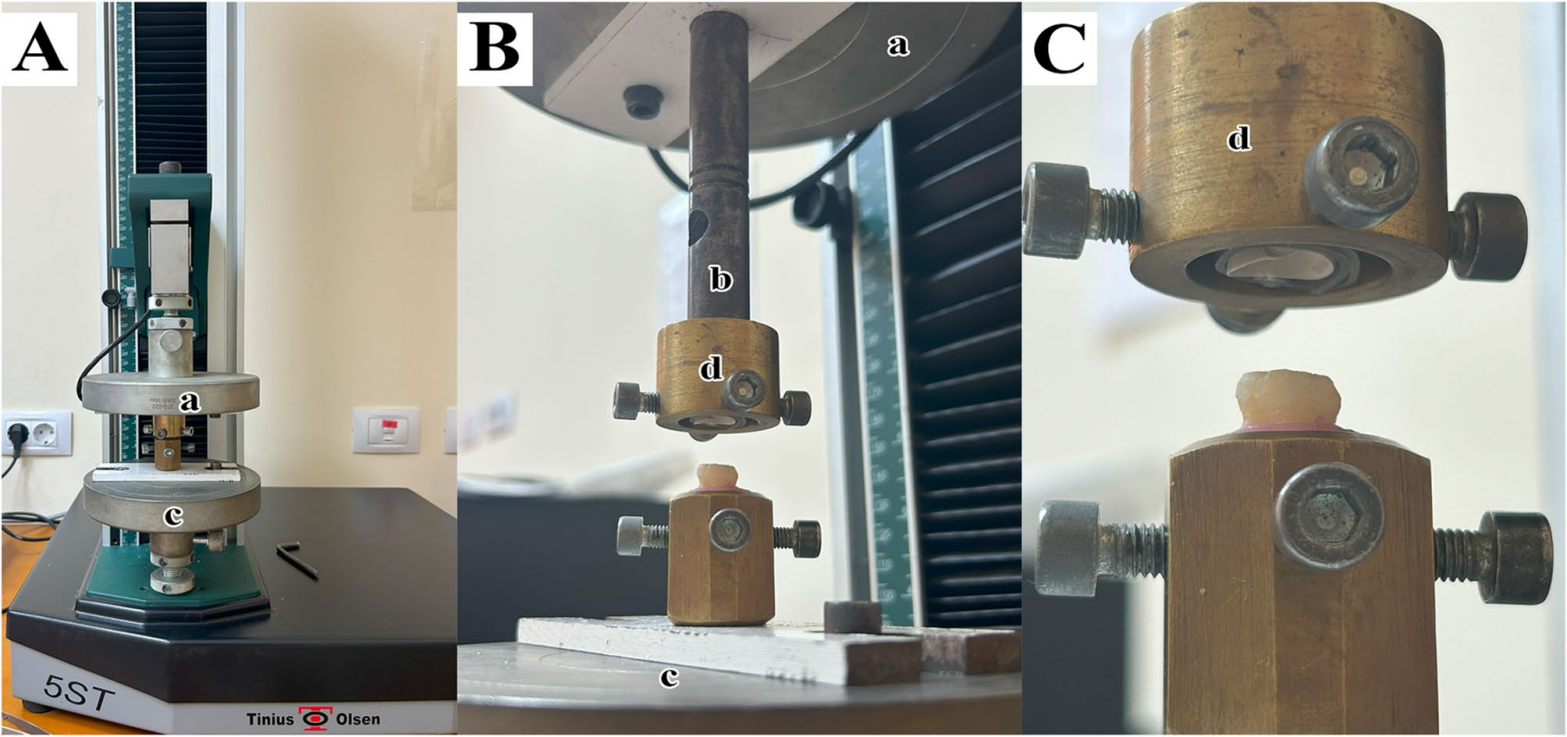
Universal Testing Machine used for retention test. **A** before load application. **B** and **C** after load application. (a; upper movable crosshead, b; upper mobile attachment, c; lower fixed base, d; customized Stainless Steel jig with 4 shoulder bolts)

After thermal aging, retention tests were conducted using a universal testing machine (5ST; Tinius Olsen, England). To stabilize each specimen, the tooth block was secured to the machine’s lower fixed base with screws. A customised Stainless Steel jig was employed to connect the crowns to the upper movable crosshead (Fig. 2). This jig included four mounting holes in which shoulder bolts were inserted to engage the crowns. A polyethylene terephthalate glycol (PETG) strip was placed between the bolts and the crown surface to act as a shock absorber and minimise the risk of fracture during testing. A dislodging force was applied along the long axis of the tooth at a crosshead speed of 1 mm/min, and the force required for crown removal was measured in Newtons (N) [43].

### Debonding failure

After dislodgment, a light stereomicroscope (B061 Olympus, Japan) at 20x magnification was used to examine the debonded surfaces [44, 45]. Debonded surfaces are divided into three categories: cohesive, adhesive, and adhesive-cohesive (mixed) debonding [46]. Adhesive failure is observed when the cement remains attached to either the tooth surface or the crown surface, but not to both. Cohesive failure occurred when cement was found on both the tooth and crown surfaces, indicating either a good bond strength between the cement and tooth/crown, or when the bond strength exceeded the cohesive strength of the cement. Mixed failure was characterised by areas of exposed tooth or crown surface, with isolated “islands” of retained luting cement remaining in other regions [46].

#### Statistical analysis

**Fig. 3:**
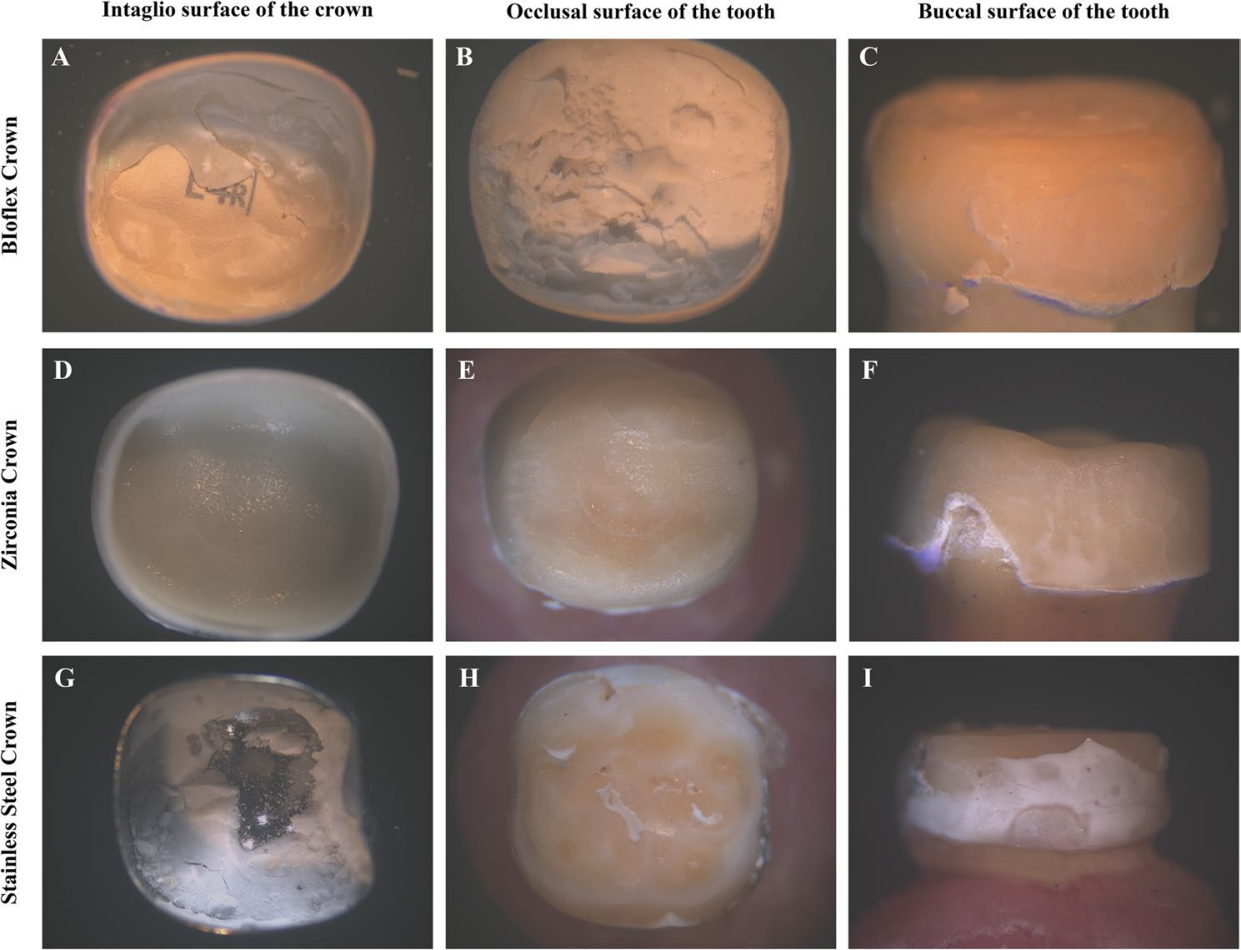
Stereo light microscope with 20x magnification images showing the debonded surfaces of the tested crowns. Bioflx Crown showing mixed debonding failure (**A**; intaglio surface of the Bioflx Crown, **B**; occlusal surface of the prepared tooth, **C**; buccal surface of the prepared tooth). Zirconia Crown showing adhesive debonding failure (**D**; intaglio surface of the Zirconia Crown, **E**; occlusal surface of the prepared tooth, **F**; buccal surface of the prepared tooth). Stainless Steel Crowns showing mixed debonding failure (**G**; intaglio surface of the Stainless Steel Crowns, **H**; occlusal surface of the prepared tooth, **I**; buccal surface of the prepared tooth)

The normality of the retention values was checked using the Shapiro-Wilk test and Q-Q plots. A normal distribution was verified; thus, the data are presented as mean and standard deviation. The mode of failure is presented using frequencies and percentages. Two-way ANOVA was used to evaluate the effects of the crown type and cement material on the retention values and the interaction between these two factors. The Pearson chi-square test was used to assess the differences in the mode of failure between the crown types. Statistical analyses were conducted using a two-tailed approach with a significance level of *p* < 0.05. Data were analyzed using IBM SPSS Statistics version 23 (Armonk, NY, USA) (Fig. 3).

## Results

Table 1 shows the retention values of the three different crown materials using the two different types of cement. According to the two-way ANOVA, both the crown material and cement type had statistically significant effects on the retention force. Crown material showed a significant effect with F = 42.42, *p* < 0.001, η²_p_ = 0.469, indicating a large effect size. Cement type demonstrated an even stronger effect with F = 186.34, *p* < 0.001, η²_p_ = 0.886, also reflecting a very large effect size. Additionally, the interaction between crown material and cement type was statistically significant, F = 6.05, *p* < 0.005, η²_p_ = 0.201, corresponding to a moderate-to-large effect.

Bioflx crowns exhibited significantly higher retention values when cemented with RMGIC (218.11 ± 34.61 N) compared to GIC (138.11 ± 30.87 N), *p* < 0.001. A similar pattern was observed for Zirconia crowns, where RMGIC resulted in significantly greater retention (131.78 ± 11.91 N) than GIC (35.50 ± 5.14 N), *p* < 0.001. However, for SSCs, no significant difference was found between RMGIC (324.11 ± 52.04 N) and GIC (307.44 ± 53.58 N), *p* = 0.513.

Within the GIC group, the SSC group demonstrated the highest mean retention (307.44 ± 53.58 N), followed by Bioflx (138.11 ± 30.87 N), and Zirconia (35.50 ± 5.14 N); these differences were statistically significant (*p* < 0.001). Similarly, within the RMGIC group, SSC again showed the highest retention force (324.11 ± 52.04 N), followed by Bioflx (218.11 ± 34.61 N), and Zirconia (131.78 ± 11.91 N), also with significant differences (*p* < 0.001).

When comparing the cements within each crown material, RMGIC showed significantly higher retention values than GIC for both Bioflx (*p* < 0.001) and Zirconia (*p* < 0.001). However, no notable differences were found between GIC and RMGIC in the SSC group (*p* = 0.513).

**Table 1:** Comparison of retention force (N) as influenced by crown and cement materials

	Bioflx *N* (*n* = 18)	Zirconia*N* (*n* = 18)	SSC*N* (*n* = 18)	*p* value
	Mean ± SD			
GIC	138.11 ± 30.87^a^	35.50 ± 5.14^b^	307.44 ± 53.58^c^	< 0.001*
RMGIC	218.11 ± 34.61^a^	131.78 ± 11.91^b^	324.11 ± 52.04^c^	< 0.001*
***p*** value	< 0.001*	< 0.001*	0.513	

^*^Statistically significant difference at *p* <0.05; different lowercase superscript letters (^a,b,c^) denote statistically significant differences between the crown materials

### Evaluation of the debonded surfaces

Table 2 compares the debonding failure rates and types across the test groups. Both Bioflx and Stainless Steel crowns exhibited 100% mixed debonding failure using different luting agents, whereas the Zirconia group showed 100% adhesive debonding failure, with the cement attached to the tooth surface.

**Table 2:** Evaluation of debonded surfaces of different crown materials

		**Bioflx** (***n***=9)	**Zirconia** (***n***=9)	**SSC** (***n***=9)	***p* value**
		n (%)			
**GIC**	Adhesive	0 (0%)	9 (100%)	0 (0%)	<0.001*
	Mixed	9 (100%)	0 (0%)	9 (100%)	
**RMGIC**	Adhesive	0 (0%)	9 (100%)	0 (0%)	<0.001*
	Mixed	9 (100%)	0 (0%)	9 (100%)	

^*^Statistically significant difference at *p* <0.05

Table 3 presents a pairwise comparison of the debonded surfaces, revealing a statistically significant difference between the Bioflx and Zirconia groups, as well as between the Zirconia and Stainless Steel groups, when either cement was used (*p* < 0.001).

**Table 3:** Pairwise comparison of debonded surfaces

**Groups**	**Compared to**	***p* value**	
		GIC	RMGIC
Bioflx	Zirconia	<0.001*	<0.001*
	SSC	1.00	1.00
Zirconia	SSC	<0.001*	<0.001*

^*^Statistically significant difference at *p* <0.05

## Discussion

The null hypothesis was rejected based on the findings, as significant differences in retention were observed among the three crown types, as well as between the luting cement subgroups within the Bioflx and Zirconia groups. However, no significant difference was found between the two cements in the Stainless Steel crown group.

Natural primary teeth were used instead of epoxy or resin dies to better simulate clinical conditions, offering a more accurate presentation of biological responses during testing. Thermal aging was performed to simulate the oral environment and to assess retention under typical clinical conditions.

SSCs demonstrated higher retention compared to both Bioflx and Zirconia crowns, regardless of the type of luting cement used. This could be explained by the precise fit of the Stainless Steel crown margins to the tooth surface, along with their ability to mechanically engage with the contours of the prepared tooth [26, 47]. These results are consistent with those of Kayal et al. [33], who compared the retention of Stainless Steel crowns and two types of Zirconia crowns cemented with GIC. They found that Stainless Steel crowns exhibited significantly greater retention than Zirconia crowns, while no notable difference was detected between the two. Similar outcomes were reported by Agrawal et al. [48], who found that after 3 months, all Stainless Steel crowns (100%) and approximately 86.67% of Zirconia crowns cemented with GIC remained on restored primary molars.

In this study, the retention of Bioflx crowns was inferior to that of Stainless Steel crowns. These findings are consistent with a recent study comparing the retention of Bioflx and Stainless Steel crowns, which reported that Bioflx crowns demonstrated inferior retention when cemented with GIC or RMGICs [26]. According to the authors, this was attributed to the inferior marginal seal of Bioflx crowns [30]. Moreover, Bioflx crowns showed superior retention compared with Zirconia crowns. This difference may be explained by the fact that Zirconia crowns require more extensive tooth preparation for placement, and their rigidity prevents post-placement adjustments, which can result in a more passive fit [48, 49]. In contrast, Bioflx crowns have a tooth preparation that closely resembles that of Stainless Steel crowns, allowing for closer adaptation and potentially better retention [21].

The RMGIC and GICs were selected for use in this study because of their superior adhesive properties [47, 50, 51]. Additionally, it is recommended that Bioflx crowns be cemented with either a GIC or RMGICs [29]. Cementation with RMGIC resulted in greater retention across all groups than that with GIC. This result is in agreement with earlier studies showing the superior retention of Stainless Steel crowns cemented with RMGIC versus GIC [52, 53]. Furthermore, Walia et al. [54]reported significantly higher retention for four types of Zirconia crowns cemented with two different RMGICs compared to GIC. A recent study also found that RMGIC provided better retention than GIC for Bioflx crowns [26]. The authors attributed this improved performance to the interaction between the cement’s HEMA components and the crown’s hybrid resin polymer, which likely facilitates mechanical interlocking at the RMGIC–Bioflx crown interface. Moreover, RMGICs exhibit a lower modulus of elasticity, higher cohesive strength, and superior bond strength to tooth structure than conventional GICs, contributing to their improved mechanical and physical properties compared to conventional GIC [55, 56].

These results contrast with those of other studies that documented greater retention in Zirconia crowns cemented with GIC than with RMGIC [32, 57, 58]. Some studies have reported that GIC achieves greater retention than RMGIC when used to cement Stainless Steel crowns [59, 60].

Stainless Steel and Bioflx crowns demonstrated a combination of adhesive and cohesive failure modes with both types of cements. This result contradicts the findings presented by Kameli et al. [53], in which Stainless Steel crowns predominantly demonstrated cohesive debonding failure. Another study [26] reported that Stainless Steel crowns predominantly exhibited adhesive debonding failure when cemented with either GIC or RMGIC, whereas Bioflx crowns demonstrated cohesive failure with GIC and adhesive failure with RMGIC. Variations in failure patterns may be attributed to differences in the abutment surfaces, scoring criteria, crosshead speed, and crown removal techniques.

The failure mode of the Zirconia crowns was adhesive, with the cement remaining attached to the tooth surface, leaving the inner surface of the crown less engaged. This may be attributable to the crown design, which lacks internal surface grooves, thereby reducing mechanical retention [4]. The findings of the present study are consistent with those of Hidayet and Ozkaya [44], who reported a high rate of adhesive failure when Zirconia crowns were cemented with both GIC and RMGIC (75% and 80%, respectively). Although our study did not include any pretreatment of the Zirconia surface, previous research suggests that surface modification, such as alumina airborne-particle abrasion, may improve retention by increasing surface roughness [61]. Moreover, the application of MDP-containing primers has been reported to enhance bonding to Zirconia [62].

This study had some limitations that should be acknowledged. Mechanical loading and other dynamic oral factors that might have influenced the retention and failure modes of the crowns were not simulated. Moreover, as an in vitro study, it lacks long-term clinical follow-up to determine the performance of the restorations under functional conditions.

## Conclusion

Stainless Steel crowns exhibited the highest retention across all types of luting cements. Bioflx crowns showed superior retention compared with Zirconia crowns when different types of luting cements were used. Additionally, the RMGIC provided greater retention than the GIC. These findings suggest that, while Stainless Steel crowns remain the most reliable option for retention, Bioflx crowns may offer a promising alternative for cases in which aesthetics are a priority in paediatric dental practice.

### Recommendations

We recommend conducting further clinical studies that incorporate masticatory simulations and long-term wear testing to better assess the durability of the Bioflx crowns. In addition, to provide a comprehensive representation of clinical performance, future studies should evaluate both the gingival response and parental satisfaction. These studies are essential to establish the long-term effectiveness and acceptance of Bioflx crowns in paediatric dentistry.

## Supplementary Information

Supplementary Material 1.

Supplementary Material 2.

Supplementary Material 3.

Supplementary Material 4.

Supplementary Material 5.

## Data Availability

Data are available from the authors upon request, but are not publicly accessible.

## Abbreviations

SSC: Stainless Steel crowns
GIC: Glass Ionomer cement
RMGIC: Resin Modified Glass Ionomer cement
PETG: Polyethylene Terephthalate Glycol
HEMA: Hydroxyethyl methacrylate
