# Supplementary material for: Retention of Bioflx, Zirconia, and Stainless Steel crowns using two different luting cements in primary molars: an in vitro study
Source: BMC Oral Health. 2025 Aug 15;25:1328. doi: 10.1186/s12903-025-06671-2 (PMC12355762; doi:10.1186/s12903-025-06671-2)
Supplement: Supplementary file 1 — Supplementary Material 1. [file 12903_2025_6671_MOESM1_ESM.pdf]

Informed Consent for Use of Extracted Teeth in Research

Study Title: Retention of Bioflex, Zirconia, and Stainless Steel Crowns Using Different Luting Cements in Primary Molars: In-Vitro Study

We are conducting a research study to evaluate the retention of Bioflex, Zirconia, and Stainless Steel crowns using two different luting materials in primary molars. The purpose of this consent form is to inform you about the use of your child's extracted primary molars for this research.

Purpose of the Study:
The primary molars to be extracted from your child will be used to evaluate the retention of Bioflex, Zirconia, and Stainless Steel crowns when bonded using two different luting materials. The findings from this study will help advance scientific knowledge in pediatric dentistry and may contribute to improvements in clinical practices related to crown retention.

Procedure:
If you agree to participate, your child's primary molars, which are being extracted for orthodontic purposes, will be used in this study. The extracted teeth will be analyzed in the laboratory, and the data collected will be used solely for the purposes of this research.

Voluntary Participation:
Participation in this study is entirely voluntary. If you choose to participate, you have the right to withdraw your consent at any time without affecting the care your child receives.

Confidentiality:
The information collected from this study will be kept confidential and used only for research purposes. No personal identifiers will be attached to the data.

Ethics Approval:
This study has been approved by the Ethics Committee of the Alexandria University Faculty of Dentistry (Approval No. 0843-01/2024, IRB No. 00010556-IORG:0008839) and was conducted following the Declaration of Helsinki.

Contact Information:
If you have any questions or concerns about this study, please contact:
Dr. Nour Morsy [01221997756] [nourgamalmorsy49@gmail.com].

Informed Consent:
By signing below, you confirm that you understand the purpose of this study, the procedures involved, and the use of your child's extracted teeth for research purposes. You also understand that participation is voluntary and that you may withdraw at any time.

Parent/Legal Guardian's Signature: ______________
Parent/Guardian name: ____________________
Date: ______________________

Researcher's Name & Signature: ___________________
Date: _________________
