# Supplementary figures and images for: Retention of Bioflx, Zirconia, and Stainless Steel crowns using two different luting cements in primary molars: an in vitro study
Source: BMC Oral Health. 2025 Aug 15;25:1328. doi: 10.1186/s12903-025-06671-2 (PMC12355762; doi:10.1186/s12903-025-06671-2)

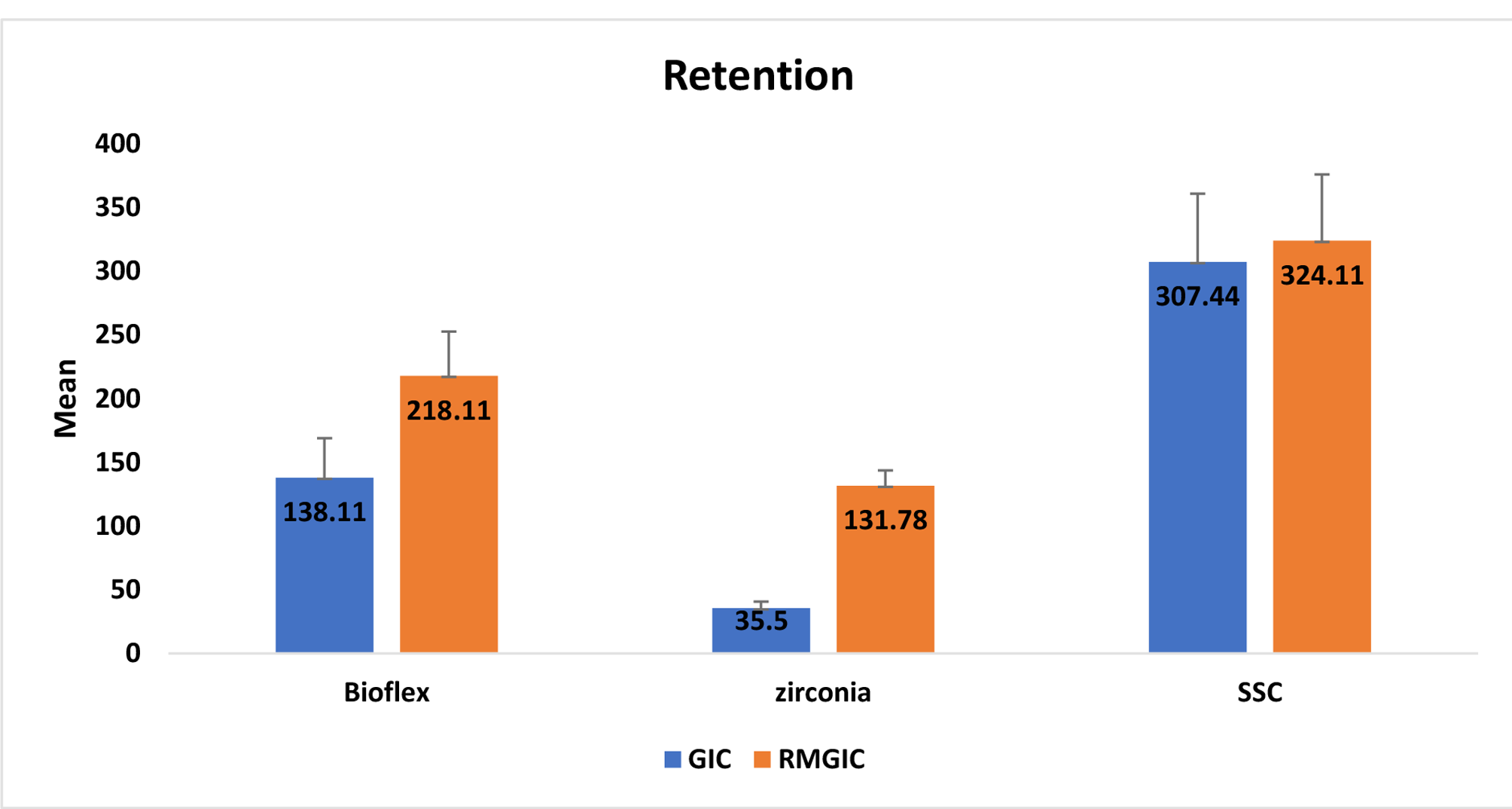

Supplement: Supplementary file 2 — Supplementary Material 2. [file 12903_2025_6671_MOESM2_ESM.tif]

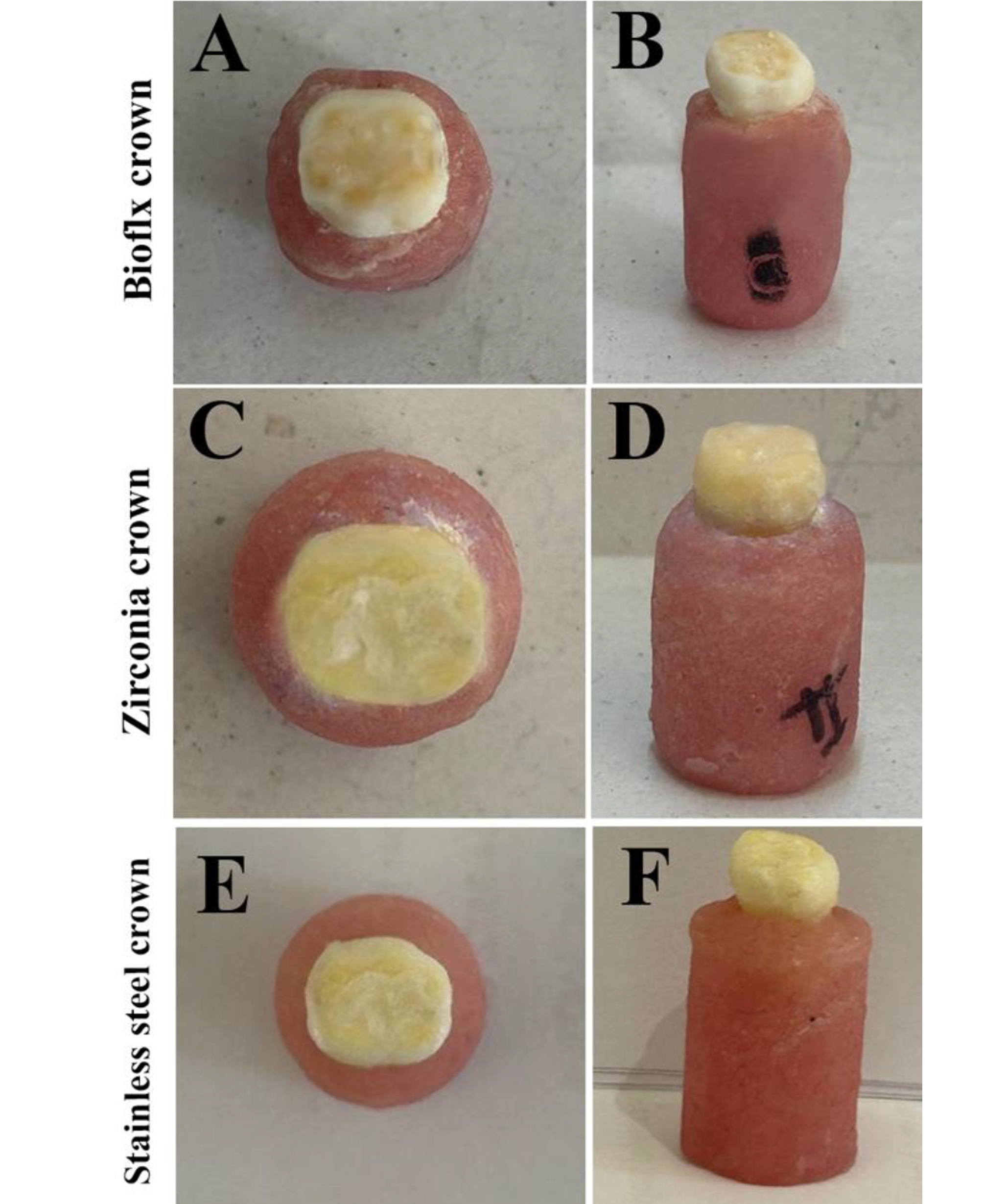

Supplement: Supplementary file 3 — Supplementary Material 3. [file 12903_2025_6671_MOESM3_ESM.tif]

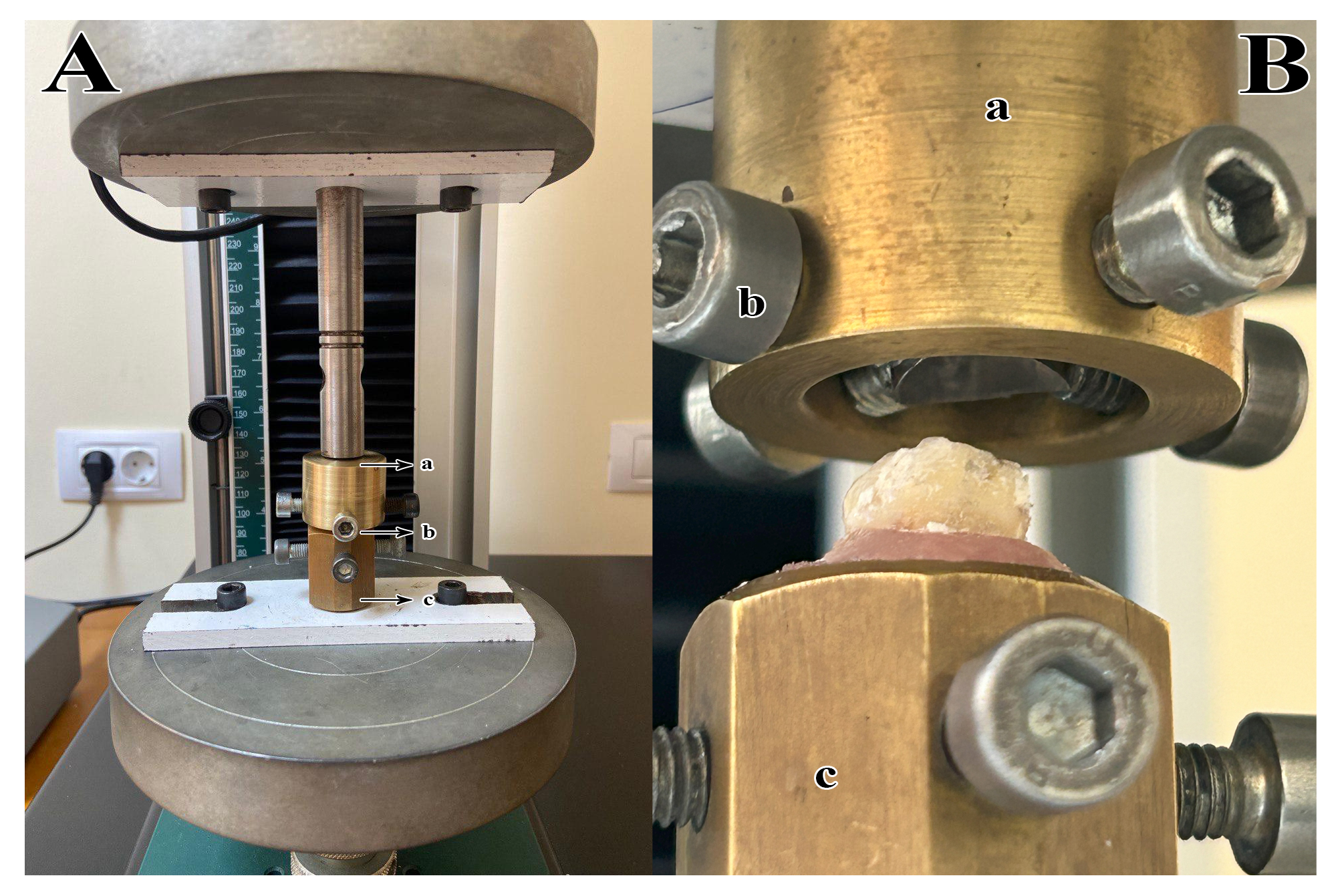

Supplement: Supplementary file 4 — Supplementary Material 4. [file 12903_2025_6671_MOESM4_ESM.tif]
