# Supplementary material for: Retention of Bioflx, Zirconia, and Stainless Steel crowns using two different luting cements in primary molars: an in vitro study
Source: BMC Oral Health. 2025 Aug 15;25:1328. doi: 10.1186/s12903-025-06671-2 (PMC12355762; doi:10.1186/s12903-025-06671-2)
Supplement: Supplementary file 5 — Supplementary Material 5. [file 12903_2025_6671_MOESM5_ESM.docx]

**SUPPLEMENTARY FIGURES LEGENDS**

**Figure (S1):** Bar chart comparing the retention between different crown types and cement materials.

**Figure (S2):** Teeth after preparation **(A;** occlusal preparation for Bioflx crown, **B;** proximal reduction for Bioflx crown, **C;** occlusal preparation for Zirconia crown, **D;** proximal reduction for Zirconia crown, **E;** occlusal preparation for SSC, **F;** proximal reduction for SSC.)

**Figure (S3):** Universal Testing Machine used for retention test. **(A)** before load application. **(B)** after load application. **(a;** upper mobile attachment, **b;** customized jig with 4 shoulder bolts, **c;** lower immobile attachment).

**Figure (S4):** Written consent form.
